# Tuberculosis Elimination in the Netherlands

**DOI:** 10.3201/eid1104.041103

**Published:** 2005-04

**Authors:** Martien W. Borgdorff, Marieke J van der Werf, Petra E.W. de Haas, Kristin Kremer, Dick van Soolingen

**Affiliations:** * KNCV Tuberculosis Foundation, The Hague, the Netherlands;; † University of Amsterdam, the Netherlands;; ‡ National Institute of Public Health and the Environment, Bilthoven, the Netherlands

**Keywords:** Mycobacterium tuberculosis, transmission, elimination, trend, research

## Abstract

This study assessed progress towards tuberculosis (TB) elimination in the Netherlands by using DNA fingerprinting. *Mycobacterium tuberculosis* strains were defined as new if the IS*6110* restriction fragment length polymorphism pattern had not been observed in any other patient during the previous 2 years. Other cases were defined as clustered and attributed to recent transmission. In the period 1995–2002, the incidence of TB with new strains was stable among non-Dutch residents and declined among the Dutch. However, the decline among the Dutch was restricted to those ≥65 years of age. Moreover, the average number of secondary cases per new strain did not change significantly over time. We conclude that the decline of TB in the Netherlands over the past decade was mainly the result of a cohort effect: older birth cohorts with high infection prevalence were replaced by those with lower infection prevalence. Under current epidemiologic conditions and control efforts, TB may not be eliminated.

## Abstract 2

Under current conditions, tuberculosis elimination may not be achieved in the Netherlands.

Reported rates of tuberculosis (TB) in the Netherlands in 2003 were 3.5 per 100,000 among Dutch residents and 125 per 100,000 among the non-Dutch. The non-Dutch are formally defined as those without a Dutch passport, but in practice they include mostly foreign-born persons. During the past 10 years, TB reports in the Netherlands declined among the Dutch (from 693 in 1993 to 531 in 2002) and remained approximately stable among the non-Dutch (at an average of 892 per year). To what extent these patterns were attributable to changes in TB transmission and to what extent to changes in the introduction of new strains from abroad or from reactivation of latent infection are unclear.

According to mathematical models, eliminating TB as a public health problem, defined as a prevalence of *Mycobacterium tuberculosis* infection of <1%, has been predicted to occur among the Dutch by 2030 if no further changes in transmission occur (1,2). However, if elimination is defined as an incidence of smear-positive disease of <1 case per million population per year (1), TB is not expected to be eliminated by that date because of transmission from foreign-source cases (2). The proportion of all Dutch TB cases in the period 1993–1998 attributable to recent transmission from a non-Dutch source case has been estimated at 12% to 20% with DNA-fingerprinting data (3). With time, an increasing proportion of TB cases among the Dutch were expected to be attributable to recent transmission from non-Dutch source cases (2). However, direct observations with fingerprinting results have not yet been used to evaluate these model predictions.

Immigration patterns in the Netherlands have varied in the past decade: large numbers of persons from countries with high TB endemicity have sought asylum in the early 1990s. In recent years, these numbers became smaller after stricter immigration laws were passed. Shifts in countries of origin of immigrants have also occurred, and some of these countries had much higher TB rates than others. Therefore, the introduction of new strains from abroad may be expected to have varied over time. Control measures, in contrast, have shown little change over the study period. TB screening is obligatory at entry and is offered every 6 months for 2 years on a voluntary basis. No routine screening for and treatment of latent infection exist for immigrants.

This study attempted to determine, by DNA-fingerprinting of *M. tuberculosis* isolates, to what extent TB trends from 1995 to 2002 were determined by changes in the introduction of new strains and by changes in ongoing transmission. We also describe the trend of TB transmission from non-Dutch source patients to the Dutch population. The combined evidence is used to assess the prospects for eliminating TB in the Netherlands.

## Methods

Patient and treatment data since 1993 were available in the Netherlands Tuberculosis Register, an anonymous case register maintained by the KNCV Tuberculosis Foundation. Reporting to the register is voluntary, but cross-matching with mandatory reports to the ministry of health on all patients who have started TB treatment suggests >99% completeness. The register includes data on demographic characteristics, clinical features, risk groups, treatment given, and treatment outcome. From January 1993 to December 2002, a total of 15,331 TB patients were registered, including those without bacteriologic confirmation.

Over the same period, 10,356 first *M. tuberculosis* isolates of TB patients were subjected to standard IS*6110* restriction fragment length polymorphism (RFLP) analysis (4). Subtyping with the polymorphic GC-rich sequence probe was carried out for strains with <5 IS*6110* copies. IS*6110* RFLP patterns were analyzed by using the Bionumerics software, version 3.5 for Windows (Applied Maths, Sint-Martens-Latem, Belgium).

Information from the 2 databases was combined; sex, date of birth, postal area code, and year of diagnosis were used as identifiers. A perfect match was obtained for 7,529 (73%) isolates and a near-perfect match for 981 (9%). Both groups were included, yielding a total study size of 8,510 (82%) culture-positive patients. Mismatches may be due to administrative errors in a database, unreliable date of birth (e.g., for some immigrant groups), or postal area code (e.g., homeless), and the exclusion of persons with identical identifiers.

TB can occur soon after primary infection or reinfection (recent transmission) or as the result of endogenous reactivation of latent infection (5). The cut-off point for separating recent from remote transmission is arbitrary: some researchers used 5 years (5–7), others 2 years (8), and others 1 year (9). We estimated the percentage of cases with identical RFLP patterns occurring within a given period after each culture-positive case with Kaplan-Meier survival analysis, as suggested by Jasmer et al. (9). The Kaplan-Meier estimate of the probability that a patient was followed by another with an identical fingerprint was 46.2% for the total study period and 33.7% for a 2-year period. Thus, of all cases followed by a patient with an identical fingerprint within 10 years, 73% were followed within 2 years. Using this information, we defined strains as new if the RFLP pattern had not been observed in another patient during the previous 2 years. Other strains were attributed to ongoing transmission. During the first 2 study years (1993–1994), judging whether strains were new was not possible; therefore, data from these 2 years were used to define new strains from 1995 onwards but were otherwise excluded from the analysis.

The observation period in which secondary cases can be observed is longer for strains introduced earlier in the study period than for those introduced later. To obtain an unbiased estimate of the trend of the number of secondary cases generated by source cases, secondary cases arising >2 years after a new strain was introduced were excluded. Thus, all patients were assigned to 1 of the following 3 mutually exclusive categories: case with a new strain, secondary case within 2 years of the introduction of a new strain, and secondary case >2 years after the introduction of a new strain. To assess the trend of transmission between Dutch and non-Dutch persons, secondary cases were attributed to a source case-patient, defined as the patient from whom the new strain was first isolated (10).

Population data by year, age group, sex, and (Dutch/non-Dutch) nationality were obtained from Statistics Netherlands (available from http://statline.cbs.nl/StatWeb) and used as denominators for incidence rates. Relative risks of TB by year of diagnosis, age, sex, and Dutch or non-Dutch nationality were determined separately for new strains and secondary cases with Poisson regression. Risk factors for the average number of secondary cases per new strain were also identified with Poisson regression (3).

## Results

Of the 8,510 TB patients with known RFLP results in the period 1993–2002, 1,580 were found in 1993 to 1994, and 6,930 in 1995 to 2002. Of the latter, 4,594 (66%) had new strains, 1,198 (17%) had secondary cases within 2 years of the introduction of a new strain, and 1,138 (16%) had secondary cases >2 years after a new strain was introduced.

**Figure 1:**
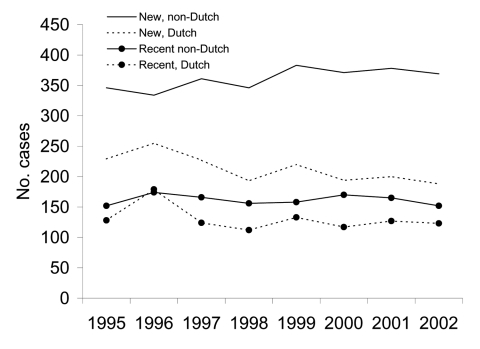
Tuberculosis incidence (new strains and strains attributed to recent transmission) among Dutch and non-Dutch in the Netherlands, 1995–2002.

The incidence of TB with new strains was on average 52 per 100,000 among the non-Dutch and 1.4 per 100,000 among the Dutch. The incidence declined over the study period among the Dutch (rate ratio per year 0.96, 95% confidence interval [CI] 0.94–0.98) and was stable among the non-Dutch (rate ratio per year 1.02, 95% CI 1.00–1.03, p = 0.06) (Figure 1). The incidence of all cases attributed to recent transmission, regardless of the duration of the cluster, was 23 per 100,000 among the non-Dutch and 0.9 per 100,000 among the Dutch. The incidence declined among the Dutch (rate ratio per year 0.97, 95% CI 0.95–1.00, p = 0.03) but not among the non-Dutch (rate ratio per year 0.99, 95% CI 0.97–1.02) (Figure 1).

**Figure 2:**
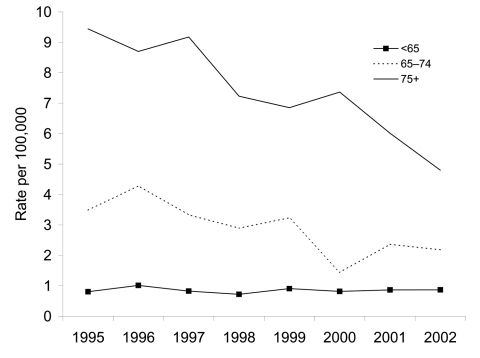
Incidence rate of tuberculosis (new strains) by age group among the Dutch, 1995–2002.

Reduction of TB incidence with new strains among the Dutch was restricted to those ≥65 years of age (Figure 2). Incidence was stable at 0.85/100,000 in the age group <65 years (rate ratio per year 1.0, 95% CI 0.97–1.03), declined from 3.5 to 2.2 in those 65–74 years of age (rate ratio per year 0.91, 95% CI 0.86–0.95), and declined from 9.4 to 4.8 in those ≥75 years of age (rate ratio per year 0.92, 95% CI 0.87–0.95). The incidence rate in the ≥75-year age group declined more rapidly than the number of cases in that age group, since the population in that age group increased from 847,000 in 1995 to 979,000 in 2002.

Of the 4,594 patients with new strains in the period 1995–2002, a total of 3,459 were found in the period 1995–2000 and could be followed up for 2 years. Of the 1,318 Dutch patients with new strains, 182 (14%) generated secondary cases at an average of 1.7 cases per cluster (1.2 Dutch and 0.5 non-Dutch) (Table 1). The average number of secondary cases generated was 0.23 per new strain and declined steeply with the age of the source case-patient (rate ratio per age group 0.74, 95% CI 0.70–0.78) (Table 1). The average number of secondary cases generated did not depend on the sex of the source patient (p > 0.5). The average number of secondary case-patients per new strain did not differ significantly over time (rate ratio per year 0.96, 95% CI 0.89–1.02).

**Table 1:** Tuberculosis cases with new strains among the Dutch, 1995–2000, and their secondary cases within 2 years

	Cases with new strains	Cases with new strains being first of cluster n (%)	Other cases in these clusters within 2 years of first case	
			Dutch	Non-Dutch
Year				
1995	229	34 (15)	52	17
1996	255	36 (14)	40	19
1997	227	29 (13)	32	8
1998	193	31 (16)	34	16
1999	220	29 (13)	31	10
2000	194	23 (12)	27	17
Age group				
<25	102	32 (31)	43	16
25–34	154	41 (27)	43	30
35–44	140	28 (20)	37	10
45–54	115	11 (10)	14	6
55–64	150	19 (13)	22	9
65–74	219	22 (10)	28	8
≥75	438	29 (7)	29	8
Sex				
Male	740	103 (14)	121	44
Female	578	79 (14)	95	43
Total	1,318	182 (14)	216	87

Of the 2,141 non-Dutch patients with new strains, 283 (13%) generated secondary cases at an average of 1.9 cases per cluster (0.5 Dutch and 1.4 non-Dutch) (Table 2). The average number of secondary cases generated was 0.25 overall, declined with age of the source patient (rate ratio per age group 0.86, 95% CI 0.80–0.92), and was lower for female than male source patients (rate ratio 0.68, 95% CI 0.57–0.81) (Table 2). The average number of secondary cases per new strain over time did not change (rate ratio per year 0.97, 95% CI 0.93–1.02).

**Table 2:** Tuberculosis cases with new strains among the non-Dutch, 1995–2000, and their secondary cases within 2 years

	Cases with new strains	Cases with new strains being first of cluster n (%)	Other cases in these clusters within 2 years of first case	
			Dutch	Non-Dutch
Year				
1995	346	39 (11)	19	68
1996	334	46 (14)	21	70
1997	361	46 (13)	19	52
1998	346	43 (12)	20	72
1999	383	63 (16)	29	77
2000	371	46 (12)	19	61
Age group				
<25	580	101 (17)	51	151
25–34	783	98 (13)	24	124
35–44	370	42 (11)	33	78
45–54	171	18 (11)	5	22
55–64	116	13 (11)	9	15
65–74	88	6 (7)	2	4
≥75	33	5 (15)	3	6
Sex				
Male	1,226	171 (14)	82	264
Female	915	112 (12)	45	136
Total	2,141	283 (13)	127	400

In clusters starting in the period 1995–2000, an increasing proportion of Dutch secondary TB cases was attributable to a non-Dutch source case as time progressed (χ^2^_trend_ 4.49, p = 0.03) (Table 3). This trend was observed not only among those cases arising within 2 years of the start of the cluster (Table 3) but also among all Dutch secondary cases, regardless of cluster duration (χ^2^_trend_ 42, p < 0.001, data not shown). The proportion of Dutch secondary cases attributable to a non-Dutch source case declined steeply with age, both among all Dutch secondary case-patients (χ^2^_trend_ 41, p < 0.001) and among those arising within 2 years of the start of the cluster (χ^2^_trend_ 27, p < 0.001) (Table 3). The proportion of cases attributed to a non-Dutch source patient was not associated with sex of the Dutch secondary case-patient (Table 3).

**Table 3:** Dutch tuberculosis cases attributed to recent transmission and diagnosed within 2 years of the start of clusters

	First case of cluster	
	Dutch	Non-Dutch (% non-Dutch first case)
Year		
1995	16	3 (16)
1996	40	19 (32)
1997	42	22 (34)
1998	32	18 (36)
1999	34	27 (44)
2000	27	23 (46)
2001	19	9 (32)
2002	6	6 (50)
Age group		
<25	29	43 (60)
25–34	46	32 (41)
35–44	32	16 (33)
45–54	27	12 (31)
55–64	24	9 (27)
65–74	34	10 (23)
≥75	24	5 (17)
Sex		
Male	124	80 (39)
Female	92	47 (34)
Total	216	127 (37)

## Discussion

This study suggests that the declining TB incidence among the Dutch in the Netherlands during the past decade has been achieved under stable control conditions. Among the Dutch, the incidence of TB attributable to new strains declined, particularly among the elderly. The incidence of TB cases due to recent transmission declined as well, a result of fewer new strains being introduced. The average number of secondary cases per new strain did not change significantly. An overall reduction in incidence of clustered cases among the U.S.-born population was also observed in San Francisco (9) and New York (11) and was attributed to improved control. In the Netherlands, we do not attribute the decline to improved control but to a cohort effect. Research may determine to what extent the reported declines in San Francisco and New York could be explained by a reduction in number of secondary cases per newly introduced strain and whether a cohort effect played a role in those settings.

In industrialized countries the annual risk for *M. tuberculosis* infection has declined steeply over the past century (12); as a result, compared to younger persons, older persons were exposed to much higher risks for infection in their youth (13). Thus, the prevalence of infection increases sharply with age. The risk for TB due to reactivation of latent infection therefore increases with age as well. Within the older age groups, this risk is now declining with each calendar year as earlier birth cohorts leave and more recent birth cohorts with lower infection prevalence enter the age group.

The incidence of culture-positive TB with new strains among Dutch persons <65 years of age was stable in the past decade, at 0.85 per 100,000 population in our matched dataset, and thus ≈1/100,000 or 10 per million if failure to match is taken into account. Of these new case-patients with a known sputum smear result, 63% had smear-positive TB. If elimination of TB as a public health problem is defined as achieving an incidence of new smear-positive TB cases of <1 per million (1), elimination is unlikely to be achieved under current epidemiologic conditions and control efforts.

Our study confirms previous predictions from a mathematical model about the increasing importance of transmission from immigrants to the Dutch population (2). The number of cases observed among the Dutch is best explained by immigrant scenarios 1 and 2 in the modeling study (2), which assume that a Dutch TB patient is 8 times more likely than a non-Dutch TB patient to infect a Dutch person.

Over time, the decline of TB incidence among elderly Dutch will become less important as the birth cohorts with a high prevalence of infection are replaced with cohorts with much lower infection rates. Contact with highly TB-endemic countries through immigrants and international travel, on the other hand, is becoming increasingly important as a determinant of TB trends in the Netherlands. This finding was shown in this study by the increasing proportion over time of Dutch patients with secondary cases attributed to a non-Dutch source patient. This finding suggests the need for further reorientation of the focus of TB control within the Netherlands towards immigrants and their contacts and reemphasizes the importance of global TB control for achieving TB elimination in countries with low incidence of this disease (14).

The separation of TB patients into those with new strains, attributed to reactivation or acquisition abroad, and secondary cases attributed to recent transmission is likely to be imperfect for the following reasons. Some strains identified as new may have represented ongoing transmission in the presence of strain evolution. Some strains attributed to ongoing transmission may represent remote transmission, particularly among the elderly (15). In this national database, epidemiologic confirmation of linkage between patients was far from complete (16). However, in a recent, more detailed study in Amsterdam, most clustered patients were found to have epidemiologic links (17). Missing data as a result of incomplete matching may have contributed to a slight underestimate of the observed clustering percentage and of the number of secondary cases per source case (18,19). However, since the matching percentage was not associated with calender year (data not shown), this underestimate should not affect the trend estimates. Some source cases may have been misclassified, in particular in large clusters. These sources of misclassification are expected to reduce the observed difference between cases with new strains and those attributed to recent transmission but do not invalidate the main conclusion that TB incidence among the Dutch was reduced mainly because of fewer reactivation cases among persons ≥65 years of age.

We conclude that the decline of TB in the Netherlands during the past decade was mainly the result of a cohort effect: older birth cohorts with high infection prevalence were replaced by those with lower infection prevalence. Contact through immigrants and international travel with countries with high TB incidence increasingly determines TB trends in the Netherlands and will prevent achieving TB elimination under current conditions. Global TB control is required to achieve TB elimination in countries with a low incidence of this disease.
